# Can #chatsafe support parents and carers beyond Australia? A qualitative study

**DOI:** 10.1186/s12889-024-19040-5

**Published:** 2024-06-11

**Authors:** Louise La Sala, Amanda Vittoria Sabo, Michelle Lamblin, Jo Robinson

**Affiliations:** 1 35 Poplar Road, Orygen, Parkville, VIC 3052 Australia; 2 Centre for Youth Mental Health, The University of Melbourne, Parkville, VIC Australia

**Keywords:** Young people, Suicide Prevention, Social media, Parents, Qualitative

## Abstract

**Background:**

Rates of self-harm and suicide are rising for young people globally and many implicate social media in this problem. To address this concern and to increase the confidence of adults to communicate safely about suicide and social media with young people, the *#chatsafe Guide for Parents and Carers* was developed in Australia. With significant uptake of the resource among Australian adults, the aim of the current study was to update and contextualise the *#chatsafe Guide for Parents and Carers* for audiences in 15 countries globally. To improve the relevance of this resource for parents and carers in these countries, the present study sought to understand the concerns held by parents, carers and suicide prevention professionals around the world about these topics and to explore the extent to which a resource such as #chatsafe would be helpful within their communities.

**Methods:**

Seven focus groups were conducted via Zoom with parents, carers and suicide prevention professionals (*n* = 40) from 15 countries. Transcribed data were coded and thematically analysed using both inductive and deductive processes.

**Results:**

Six themes are reported: (1) Two scary ‘S’ words; (2) Country and culture impact who talks (or is silent) about self-harm and suicide; (3) The need for a protective social ecosystem; (4) #chatsafe is a tool that can help parents, carers and young people worldwide; (5) #chatsafe should consider local context and end users to improve its relevance for parents and carers worldwide; and (6) A range of marketing and dissemination strategies are needed to reach adults with #chatsafe information. Findings of this study informed the update and contextualisation of the *#chatsafe Guide for Parents and Carers* for adult audiences in 15 countries.

**Conclusions:**

The findings from this study underscore a universal need for psychoeducation initiatives that provide adults with the skills and knowledge to support the mental health of young people, both online and offline, and that resources like #chastafe can play an important role in providing reliable information about these topics to adults across a range of cultures and contexts.

## Background

Suicide is the fourth leading cause of death for young people globally, with adolescents in Low- and Middle-Income Countries (LMICs) accounting for an estimated 88% of suicide deaths in this age group [1, 2]. Despite an observed decline in population suicide rates in many parts of the world over the past two decades [3], rates continue to rise for young people under 25, with particularly sharp increases in suicide mortality among adolescent girls [4–6].

The risk factors for self-harm and suicide are multifaceted, yet many question the impact of social media. Most widespread are concerns about social media use contributing to negative mental health outcomes for young people [7, 8], including self-harm and suicide risk [9–12]. Social media has been associated with increases in self-harm behaviour and suicidal ideation, particularly among populations with existing risk factors [12]. Exposure to self-harm- or suicide-related content online is also thought to play a role in suicide contagion or imitative behaviours for some young people [13]. While social media may present risks to certain groups of young people, there are methodological limitations in much of the literature in this area (e.g., reliance on self-report and cross-sectional designs) that undermine generalised claims of social media increasing psychological distress among young people more broadly [14]. Indeed, a growing body of literature documents the ways in which some young people perceive social media to be an important component of their wellbeing, such as through connection with others and providing an often-utilised avenue of help-seeking [15, 16].

The overrepresentation of studies correlating social media use with poor mental health outcomes for young people is likely amplifying common concerns held by many adults and often captured by public sentiment [17–19]. Parents and carers of young people in Australia from a range of backgrounds almost unanimously believe that the internet and social media expose young people to danger or risk [20, 21], and tend to perceive their children as being unsafe online far more often than young people report feeling unsafe themselves [22]. The features of social media that young people tend to appreciate, such as social connection with peers and like-minded others, constant access to broad social networks, and exposure to new information and ideas [23, 24], correspond with some prominent concerns about youth social media use held by adults, including contact with strangers, excessive screen use, and exposure to harmful content or information [25].

This presents an interesting time for parents today, many of whom feel that parenting is more difficult now than it has been in previous decades, largely due to the impacts of technology and social media [26]. Globally, parents report that they are finding it more difficult to monitor their young person’s online activities, and that trust in their child’s ability to act responsibly online is decreasing [27]. At the same time, teenagers report being less likely to turn to their parent for help than they would a peer, if confronted with a negative online experience [22]. Such findings indicate that topics relating to online safety, social media, and online behaviour are not well communicated between parents and young people. When conversations about these topics do take place, parents often describe them as overwhelming and fatiguing [22]. It is clear that digital technologies have prompted profound and widespread changes in how we communicate, find information, share our experiences, and seek community and support around the world [28], and have become a necessary point of consideration in modern parenting.

### #chatsafe

Recognising young people’s widespread use and perceived benefits of digital technologies, emerging interventions have sought to capitalise on the reach and accessibility of social media to improve public health outcomes for this population (e.g., [29, 30]). One such approach is the #chatsafe intervention, which comprises evidence-informed guidelines for young people aged 12–25 and a youth co-designed social media campaign that delivers age-appropriate suicide prevention psychoeducation to young people entirely via social media [30].

The #chatsafe guidelines [31] are accompanied by a suite of adult-facing resources intended to equip parents, carers and educators with the skills to support safe online communication about self-harm and suicide [32, 33]. In 2021, the guidelines were adapted and translated for young people in ten countries worldwide [34], with social media content related to these global versions reaching more than one million young people in the related countries within a 6-week period. Edition two of the guidelines and associated resources were developed in 2022 and 2023 [31–33, 35] to include information about self-harm and reflect current social media trends. Across all iterations of #chatsafe resources, the *#chatsafe Guide for Parents and Carers* [32] has consistently received the most uptake, with more than 186,000 downloads across all versions of this resource between August 2021 and March 2024. The Australian version of this resource has previously been translated into 11 languages reflecting the most common culturally and linguistically diverse populations in Victoria, Australia, and is currently being evaluated as a training program for parents and carers of high-school aged young people in Australia. Despite this high uptake and application in Australian settings, the *#chatsafe Guide for Parents and Carers* had not been contextualised or translated for international audiences until now.

The primary objective of the current study was to develop new versions of the *#chatsafe Guide for Parents and Carers* for audiences in 15 countries across various global regions. To ensure that the updated guide for parents and carers adequately reflected the needs of end-users in these countries, the following research questions were examined:

1. What concerns do parents, carers and mental health organisations in different parts of the world hold about youth self-harm, suicide, and social media use?
2. To what extent could a resource such as the *#chatsafe Guide for Parents and Carers* support international parents, carers, and mental health organisations that work with families?

## Method

### Study design

This was a qualitative interview study that utilised focus groups with parents, carers and suicide prevention professionals from local mental health organisations in 15 countries (Brazil, Canada, Chile, Denmark, India, Ireland, Japan, Nigeria, Philippines, South Africa, South Korea, Sweden, Thailand, The United Kingdom/UK, and The United States/USA). The study was conducted by researchers based in Melbourne, Australia, and received ethics approval from The University of Melbourne Human Research and Ethics Committee (ID: 22728). This study has been reported in line with the Consolidated Criteria for Reporting Qualitative Research (COREQ; [36]).

### Research team and reflexivity

All researchers listed on this study have been trained in qualitative research methods, have expertise in youth suicide prevention, and were involved in the development of the #chatsafe guidelines and resources. The researchers who facilitated the focus groups and undertook data coding and analysis (LLS and AVS) were both white Australians who identify as female, and both have professional experience in research activities focused on youth suicide prevention, community mental health interventions, and gatekeeper training. Some participants were known to the research team prior to their involvement in this study through pre-existing professional relationships. All participants were informed of the researchers’ aim to develop updated and contextualised versions of the *#chatsafe Guide for Parents and Carers* for each country included in the study. It was expected that participants would have varying degrees of prior knowledge and exposure to the #chatsafe program of work. The facilitators made efforts to reduce any perceived pressure to provide desirable feedback during the focus groups by monitoring their tone, verbal language and body language, and engaging equally with strengths and limitations of the #chatsafe resources when prompted.

### Sample and recruitment

The research team established partnerships with leading mental health or suicide prevention organisations in the 15 countries involved in this study. Eligible individuals from these organisations were invited to take part in this study via email and then asked to advertise the study more broadly to parents, carers and professionals within their networks. This approach combined purposive and snowball sampling to achieve a sufficiently diverse and knowledgeable sample group. All prospective participants were required to complete an online consent form and demographic survey.

Individuals were eligible to participate if they were: at least 18 years of age and proficient in written and spoken English; and either currently employed in a government department or policymaking role with responsibility for mental health, youth, or online safety; or currently employed in a mental health or suicide prevention organisation in a clinical or research capacity; or a parent/guardian/carer/family member of a young person aged 12–25. There were no additional exclusion criteria.

In total, 42 adults from 15 countries attended the focus groups, and 40 participants consented for their data to be included in the study. Two participants provided verbal consent during the recorded session but failed to complete the online consent form and were therefore excluded.

### Data collection and analysis

Six focus groups were conducted in English online via the Zoom video conferencing platform in March 2023, each ranging from 60 to 90 min. The facilitating researchers (LLS and AVS) conducted these sessions in private spaces and participants joined the online meeting from residential or professional settings, some of which were public spaces (e.g., open-plan office). Where possible, focus groups were arranged based on global region or time zone to reduce scheduling conflicts, though participants were able to attend whichever session suited their availability: Session 1 (Asia Pacific, *n* = 14), Session 2 (Europe, *n* = 8), Session 3 (Americas, *n* = 6), Session 4 (Africa, *n* = 5), Session 5 (Americas, *n* = 4), Session 6 (Denmark and Philippines, *n* = 5). During each focus group a facilitator delivered an introduction to the #chatsafe program of work, and participants were then invited to take part in a structured discussion about their concerns relating to youth self-harm, suicide and social media use, and their thoughts on the *#chatsafe Guide for Parents and Carers* (also referred to as ‘the resource’) and its relevance (or not) for their country. Three groups of questions guided the discussion: (1) When it comes to these topics generally: what are your concerns when it comes to suicide, self-harm and social media? (2) When it comes to these conversations with young people in your region: are suicide, self-harm and social media topics that parents and carers (or other key adults) communicate about with young people? Are these conversations had in your community? What are the barriers/challenges? (3) How can #chatsafe for parents and carers support you? What are your thoughts on the #chatsafe for parents and carers resource? How can it support parents and carers from your region? What else should be included? Is there anything missing?

Audio recordings were transcribed verbatim by a third-party transcription service. Transcribed data were coded and analysed by two researchers (LLS and AVS), using thematic analysis guided by principles outlined in Braun and Clarke [37]. Frequent research meetings were used to discuss and iteratively refine the coding output, and ongoing dialogue allowed for reflective development and refinement of themes. To address the first research question, relevant data were coded and analysed using an inductive approach to reflexively develop meaning contextualised within the researchers’ prior knowledge and expertise. To inform updates to the *#chatsafe Guide for Parents and Carers* in a practical way, data relevant to the second research question were coded and analysed deductively. Broad categories of possible feedback relevant to the second research question were discussed and determined by the researchers after familiarising themselves with the data, and centred on: (1) perceived relevance or usefulness, (2) opportunities to improve relevance or usefulness, (3) marketing and dissemination. Coded text was extracted into a data matrix in Microsoft Excel, to further develop theme headings and identify illustrative quotes. Where possible, findings from this study were incorporated into the development of edition two of the *#chatsafe Guide for Parents and Carers*, with 15 global versions in 11 languages produced and contextualised for audiences worldwide. To preserve fidelity to the evidence-based #chatsafe information [31, 35], feedback that conflicted with or deviated substantially from information in the #chatsafe guidelines was not implemented. However, each global version of the resource incorporated a one-page summary of key findings relevant to the respective country, as well as localised information about support services and other resources. All versions of the resource can be found on the #chatsafe website (see: [38]).

## Results

### Participants

Of the final sample of 40 participants, 25 identified as female (62.5%) and 15 identified as male (37.5%). The average age of participants was 44 years (range: 19–75). All 15 countries were represented by at least one participant. Seventeen participants (42.5%) selected that their primary source of expertise was as a parent or carer of a young person aged 12–25, and 14 of these participants also worked at a mental health organisation in a suicide-prevention-related role. Similarly, many participants who selected that their primary source of expertise was “professional” also disclosed experiences with their own children or young people in their care during the focus groups, indicating that most participants had dual sources of expertise. Those nominating themselves primarily as suicide prevention professionals (*n* = 23, 57.5%) held a wide range of positions at various levels within their organisation (e.g., volunteer, project manager, head of communications, psychologist, professor, chief executive officer), and duration of their current role ranged from one month to 37 years. A range of government bodies, non-government organisations (NGOs) and academic institutions were represented.

All but three participants reported using one or more social media platform (*n* = 37, 92.5%), with the majority of participants using three or more platforms (*n* = 25, 62.5%). The most common platforms used included Instagram (*n* = 28, 70%), YouTube (*n* = 28, 70%), Facebook (*n* = 25, 62.5%) and Twitter (*n* = 21, 52.5%). When asked about whether they had ever communicated on social media about self-harm or suicide, or encountered and/or engaged with content on these topics, almost two-thirds of participants responded “yes” (*n* = 25, 62.5%).

### Research question 1

#### Two scary “S” words

Irrespective of participants’ primary source of expertise, there was substantial fear or concern about suicide and social media, and those fears were exacerbated in relation to young people. Concerns about these topics seemed to be underscored by a perceived lack of knowledge and confidence with suicide and social media as both standalone and overlapping topics.

##### Adults lack the knowledge to talk about suicide

A consistent idea that emerged in all focus group discussions was that adults do not know how to talk about self-harm or suicide. Fear and avoidance of these topics were attributed, by some, to prominent myths that talking about suicide is inherently risky and will put the idea in a young person’s mind. Knowledge to combat these misperceptions was described as almost universally lacking, with adults around the world being perceived as simply not having the tools or confidence to communicate openly about self-harm or suicide with anyone, let alone young people. Irrespective of the degree of suicide stigma or literacy participants perceived within their country, these conversations were widely described as feeling uncomfortable, risky, difficult, and avoided.

*“I think a lot of parents are afraid to bring up this subject matter – suicide and self-harm – one, because of their own ignorance maybe, or lack of knowledge about it, and two, in case they’re triggering something with their children.”* – P21, Ireland.

*“We have this conception that when you talk about suicide, we facilitate someone who wants to and didn’t have the chance or the opportunity. So, we don’t communicate, we don’t talk.”* – P36, Brazil.

Indeed, participants from some countries (e.g., Sweden, Thailand) identified that young people are much “*more comfortable and they have less stigma in talking about these issues*” (P18, Sweden) than adults, possibly because of exposure to online information and experiences related to self-harm and suicide. In these circumstances, the avoidance and discomfort from adults appeared to stifle open communication, leaving young people to navigate their thoughts and feelings about these topics through informal channels of support such as social media and peer groups.

*“…[young people] have 24 [hour] access to groups and discussions about mental wellbeing, self-harm, and suicide, but there is a lack of conversation from the adult world. And I think here is the biggest problem.”* – P18, Sweden.

##### Adults feel left behind by social media

Despite the overwhelming majority of participants reporting that they utilise at least one social media platform in their daily lives, many perceived themselves and other adults as lacking the digital literacy and knowledge required to engage confidently in conversations about online safety with young people. Some participants who were parents expressed feeling out of touch or “*like a dinosaur in terms of social media*” (P20, Ireland), and that the skills required to navigate constantly evolving platforms, let alone support their young person to do so, were outside of their expertise.

*“I think although my generation have a better concept of social media than my parents did, I think a lot of parents still feel left behind because it’s developing quite rapidly, and new social media are coming all the time.”* – P38, Denmark.

The number of platforms that young people engage with and the near constant access they have to their online social worlds were sources of concern for parents, carers and professionals alike. Adults’ lack of knowledge about social media was also perceived to reinforce their anxieties about the perceived risks associated with digital platforms and young peoples’ susceptibility to these risks.

“*There’s always heightened anxiety among parents – that’s a universal. And I think there’s a lot of anxiety among parents about social media use, period. Whether it’s destructive, around suicide or self-harm, or not.”* – P34, USA.

While these concerns were evident across all focus groups, some participants did acknowledge the importance of online social connection for young people and dismissed sweeping judgements about social media. Weighing the possible benefits of online spaces against implications for in-person interactions presented a recognition of the necessity of nuance in conversations about helpful versus harmful social media use.

*“While it can be a source of connection for some, and there’s a value there, it really has almost taken the place of face-to-face interaction in human connection. So, there’s pros and cons, but there’s a real concern that overuse – that’s another piece – how can you not only use these platforms responsibly, but be moderate in your amount of use or your level of use. Without saying ‘social media is bad’ – that’s a silly thing to say – but what are the parameters that make for healthy use of social media platforms?”* – P34, USA.

##### Adults worry that the combination of suicide and social media is particularly unsafe

Concerns about suicide and social media as independent topics were almost universally present, however, more specific fears emerged when considering young people’s possible exposure to self-harm- and suicide-related content on social media. Indeed, some concerns about social media in itself stemmed from anxieties related to the types of unregulated content (e.g., graphic or violent self-harm or suicide trends), potentially predatory behaviour (e.g., encouragement of self-harm and suicide in individuals at risk), and harmful information that young people may be exposed to online.

*“I think probably my biggest concern is – and the concern of families that we speak with – is the type of information that young people can access online that can be destructive…. You know – ‘if I want to die by suicide, here are the 10 things Joe Blo’s going to tell me that I can do in order to die by suicide.’*” – P34, USA.

Examples were given of harmful self-harm- and suicide-related videos, memes, and online “*games that teach you how to take your life*” (P39, Philippines). The scale and vast reach of unsafe or unregulated content was especially alarming, and tapped into fears about suicide contagion or imitation, especially in parents. However, some professionals were concerned that unsubstantiated trends garnered more attention than is warranted due to panic and “*misinformation around incidents*” (P16, Ireland) being spread among concerned parents and school communities.

Although these concerns were broadly shared, there were a few accounts wherein some participants described feeling a great degree of confidence in the young people in their lives to engage safely with sensitive topics online. This confidence seemed to be the outcome of open and ongoing dialogue about online safety, and development of mutual trust in the relationship. When asked whether they were concerned by young people talking online about self-harm and suicide, one participant replied:

*“Not when my lad’s concerned, to be honest, because he’s pretty resilient – pretty sensible, he understands that there are sources of support out there.”* – P15, UK.

#### Country and culture impact who talks (or is silent) about self-harm and suicide

Stigma and cultural beliefs about mental health emerged as the dominant factors that influence how conversations about these topics are discussed around the world and with whom. In almost every focus group, participants talked about mental health stigma being “*alive and well*” (P28, Canada), and more so when it comes to suicide. Many participants conflated mental health with suicide in these discussions, and the following results reflect this.

Certain cultural beliefs were described as powerful barriers to open communication and help-seeking, with those experiencing mental health challenges or thoughts of suicide being subjected to harsh judgments within their families or communities: “*a sign of weakness*” (P18, Sweden), “*attention seeking*” (P7, India), being “*too soft*” (P33, Nigeria), or being “*cursed*” (P30, South Africa). One participant described their country as having “*a lot of history of hiding people that are mentally unwell*” (P18, Sweden), contributing to a legacy of harmful beliefs and stigma around mental health that are persistent across time.

Participants described expectations to conceal struggles and ‘perform wellness’ for the sake of honour, family and community coherence as being created and reinforced by cultural beliefs around how people should conduct themselves. They attributed these as a cause for open dialogue being restricted.

“*Children are expected to perform. By performing, that also includes they are well mentally*.” – P5, Thailand.

“*…talking about my depression to my parents or my friends makes me feel that I am very weak or vulnerable…I think those cultural beliefs and standards, that we should all be good enough for everyone and we should look good enough for everyone, might be one of the problems that makes us hesitant to talk about those topics.”* – P11, South Korea.

Shame associated with mental health struggles was described as being upheld by cultural beliefs that children “*cannot shame the family*” (P5, Thailand). This was alleged to result in “*dismissal of young person’s distress*” (P7, India) to avoid their families being perceived in an undesirable way by association. In a similar vein, participants from Nigeria and Thailand who facilitate support services described instances where young service users were referred on to a mental health professional or doctor, only to have their parents deny access to professional treatment in order to avoid shame or judgement.

“*The parents are always saying that ‘you don’t need to go’ because it’s the shame or whatever for the parents also. Even though the young people, they know themselves that they have to go, but the parent always say that ‘you don’t take any medicine that psychiatrists give you’ or ‘you don’t need to go.*’” – P6, Thailand.

These factors were also perceived to inform how conversations occur after someone has died by suicide. One participant described situations where parents “*openly deny that their children took their lives”* (P39, Philippines), while families elsewhere may attribute a suicide death to other causes like substance use or “*being cursed*” (P30, South Africa).

Age was believed to influence who perpetuates harmful cultural beliefs and stigma, with many participants describing a sense of comfort and willingness among young people to discuss self-harm and suicide, versus discomfort and denial on the part of adults.

“*Parents will say – or carers – they’re not really openly willing to talk about these topics, even if young people are really expressing themselves.”* – P30, South Africa.

Exacerbating this disconnect was a sense that it is not typical for parents in some countries to be emotionally close or open with their children. Cultural differences in parenting and ‘family time’ were perceived to result in parents having “*very little insight about their children or the children’s psychology in general*” (P11, South Korea), creating more closed relationships where sensitive topics are avoided. In India, hierarchical family systems were put forward as a contributor to similarly closed relationships wherein “*talking about your inner thoughts and distress may not be even acceptable a topic of communication*” (P7, India).

Broader societal factors such as a lack of mental health education in schools and socioeconomic disparities among the population were described as contributing to adults lacking “*the information to start a communication with young people*” (P36, Brazil) about self-harm or suicide. In some countries, this was compounded by criminalisation of suicide at a national level, which was described as creating fear among the population “*that the moment you talk about it, you are already [breaking any law]*” (P33, Nigeria).

Despite these complex and intersecting factors that may create barriers to open communication about self-harm and suicide among families and communities, there was a distinct sense of recognising how far some societies have come in terms of mental health knowledge and awareness, and indeed, hopefulness for a future where suicide “*can be discussed without fear and in a safe place*” (P39, Philippines). This sentiment was most often present in participants from High-Income Countries (HICs) and/or Western countries (e.g., Ireland, UK, Sweden, Canada, USA), many of whom perceived a notable improvement in mental health stigma in recent decades, especially among younger generations.

“*I think yes there’s still stigma, but I think we are globally working towards making ourselves even more comfortable with it. I can say for myself, it’s an uncomfortable subject to talk about but that doesn’t mean we can’t do it*.” – P25, Canada.

#### The need for a protective social ecosystem

Although many participants acknowledged the important role of parents and carers in being the first point of contact for topics impacting young people, they also felt that parents and carers cannot provide effective support alone. The role of other key adults in a young person’s life such as educators or extended family members were discussed, with calls for consistent mental health education and suicide prevention training to be provided to all adults who may interact with young people.

“*…adults interact with children in other settings, like on the football pitch, and in the school, and…that audience might be just as important, ultimately, in terms of being able to support maybe slightly more vulnerable children and young people*.” – P22, Ireland.

At the highest level, governments were described as playing a critical role in supporting NGOs and mental health organisations that operate within communities. Participants also shared a desire for tighter regulation of online service providers to reduce circulation of unsafe content and risk of harm. The recent establishment of an online safety commissioner in Ireland to prioritise development of online safety codes relating to self-harm and suicide was presented as a positive example of regulation in this space, however, there were still calls for firmer action by companies to “*prevent some of the content being shared on their social media platforms*” (P18, Sweden).

Community contexts such as schools, sports clubs, hobby centres, religious groups and libraries were discussed by many participants as central spaces that can support young people, their peers, and their families. One participant described how their organisation leveraged school and community events to gently introduce conversations about mental health into communities with high mental health stigma:

*“…If we say ‘we have a mental health topic in a school’, the mothers don’t come. They will not come. They will not come but if you say ‘we’re going to do some fashion trends and what is fashionable and what mothering today is all about’ and we bring in the idea, the thoughts about social media and all of that, they are very open. They open up but we have got to handle our parents differently*.” – P39, Philippines.

In-person mental health services and community outreach were perceived to play a particularly important role in supporting communities in areas with reduced access to digital technology, limited mental health vocabulary, and high stigma (e.g., South Africa, Nigeria). Having these services localised and operated by members of the target community who “*come from the same environments*” (P29, South Africa) as young service users was described as key to their effectiveness.

Despite concerns about social media discussed previously, a few participants felt it was important to recognise the role of online spaces as an invaluable mode of peer-to-peer support and help-seeking. This was perceived as particularly relevant for young people who may have few in-person supports, groups of young people grieving a suicide death in their community, or those who experience substantial cultural barriers to communicating about these topics in-person. However, some simply acknowledged that young people may prefer to communicate and seek information online irrespective of whether they have access to other supports, and that there is value in utilising these spaces for prevention and advocacy activities because *“[youths] are more on social media*” (P33, Nigeria).

“*Those who are vulnerable and those who [have little offline] communication is likely to lean on to their online friends and their online community.”* – P9, South Korea.

“*I think a lot of young people being vulnerable actually see these kinds of groups and so on, because maybe they feel safer here than talking to their parents.”* – P38, Denmark.

Weaving together these different contexts, one participant described a need for “*continuity of information*” (P19, Ireland) across families, communities and online settings, enabling young people to be supported wherever they are. Creating communities that are empowered with the tools and knowledge to communicate about self-harm and suicide, online and offline, was perceived to also benefit parents in supporting other parents, while enabling young people to safely provide support to their peers and/or seek support from a range of knowledgeable adults. This kind of protective ecosystem that includes mental health and suicide-prevention education across the broad spectrum of society (e.g., schools, sports clubs, medical centres, community groups), was believed to be a necessary part of supporting a “*resilient population*” (P36, Brazil) and better outcomes for young people.

### Research question 2

#### #chatsafe is a tool that can help parents, carers and young people worldwide

In every focus group, participants expressed a need for broader mental health education, suicide prevention information and resources to improve digital literacy in adults worldwide. Irrespective of country or contextual factors, there was a sense that resources like the *#chatsafe Guide for Parents and Carers*, as well as the #chatsafe materials more broadly, have something to offer parents, carers and families in line with these needs.

“*I think that this resource would definitely be valuable as we can all see, because again the more comfortable I think we are as parents and carers with this subject, potentially the more comfortable our children will be asking us, right?”* – P25, Canada.

The limited mental health vocabulary and high rates of stigma described by participants from South Africa led them to favour digital technologies as a positive opportunity for young people to connect with others about self-harm and suicide, while bypassing cultural barriers to open in-person communication. Guidance for safe online communication about self-harm and suicide was described as particularly relevant for young people in these communities.

Novel guidance about online self-harm and suicide games, pacts, challenges, and hoaxes, introduced in edition two of the #chatsafe guidelines [31], was described as being particularly relevant to current global trends, which represent a challenge for suicide prevention efforts. The inclusion of this new information was viewed as a valuable way of counteracting misinformation and parental anxiety about these topics.

“*I think it’s great to see the extension to look at content on games and challenges; I think, professionally, that’s always a kind of space that we struggle with…I think from a parent’s point of view, these things are maybe not understood, or they’re misunderstood a little bit*.” – P16, Ireland.

Additionally, information about why and how to use safe language when talking about self-harm and suicide was particularly relevant to the concerns of families and professionals. Evidence-informed information presented in the *#chatsafe Guide for Parents and Carers* was perceived as one part of the solution in the challenge of combatting “*the really negative and destructive and inflammatory and incendiary language*” (P34, USA) that can proliferate suicide-related harm online. The goal of #chatsafe to improve safe online communication about self-harm and suicide was recognised as an important objective.

#### #chatsafe should consider local context and end users to improve its relevance for parents and carers worldwide

While the value of #chatsafe materials for families and communities around the world was widely endorsed, opportunities were identified to improve the relevance and accessibility of the *#chatsafe Guide for Parents and Carers* for adults in each country. Notably, most participants expressed a desire for country-specific versions of the resource incorporating new information that contextualises the resource to their local context and culture.

“*It’s mostly a cultural thing, I think, in comparison to the West, where parents are probably more open-minded with regards to their children’s performance. I think this is one thing that is a concern and maybe it’s something that we can discuss, about country specific approach to help young people with #chatsafe.”* – P5, Thailand.

Participants from LMICs (e.g., India, Thailand, South Africa, Nigeria) and/or non-Western countries (e.g., Brazil, Chile, Japan, South Korea) commented that the resource needed to recognise country-specific factors that may impact vulnerability to risk (e.g., caste, class and religion in India), and cultural factors that may influence mental health stigma and (lack of) open communication among family members. One participant felt that future iterations of #chatsafe or similar resources should also include more “*scaffolding*” (P37, Chile) of mental health information for countries with poor mental health literacy; a sentiment that garnered agreement within their focus group. Suggested factors to contextualise the resource for these countries are detailed in Table 1.

**Table 1:** Suggested factors to contextualise the resource for LMICs and/or non-Western countries

Contextual factors	Illustrative quote
Caste, class, sexuality, religion, language	“In a country like India, layers of caste or class or even sexuality, religion, language produce certain privileges and vulnerabilities, and it’s also important to have something spelled out very clearly about that.” – P7, India
Religious communities	“At least here in Brazil, what I see is that maybe it needed a special communication about religious community. Because a lot of stigma around some religions and the – even for the suicide of religious leaders. Maybe this is a topic that could be addressed differently to parents to talk with the teenagers; even if they have a faith or a religion, how they can talk about suicide freely.” – P24, Brazil
Cultural barriers to open parent/child dialogues about mental health	“It would be helpful to add to the [first] half of the guideline what to do when parents and children do not have the habit or relationship to share about their feelings with each other. The second is, it would also be helpful to include information on how to respond to rejection by children when they try to talk with their own children about suicide or self-harm. This is because I believe that Japanese parents would be concerned about their children rejecting their requests for dialogue about suicide and self-harm from their children.” – P4, Japan
Family honour, community shame, pressure to perform	“Then some specific barriers that could be experienced by parents or caregivers from Asian culture, like family honour, community shame. We are not just worried about individual opinions, but also larger cultural…barriers or common thoughts also, that young children should be successful, performing very well, all of that also.” – P7, India
Criminalisation of suicide	“…apart from our organisation, we have a lot of other organisations [actually fighting] about it because I think that suicide is presently a criminal offence, in Nigeria…” – P33, Nigeria
Lack of mental health vocabulary	“…South Africa’s very culturally rich in that we have 11 various cultures – and in many of those cultures and of the languages…there isn’t vocabulary around mental health, suicide, depression and so on.” – P29, South Africa
Poor mental health literacy	“…[in] Latin America, we are more poor and we don’t have some resources for manage [mental health] information, to understand that information in the correct way, so I think that have a more scaffolding first.” – P37, Chile

Participants from HICs (e.g., Ireland, UK, Canada) more often requested additional content to validate parents’ concerns about their young person’s social media use, and information to improve adults’ digital literacy. Opportunities to improve the usefulness of the resource for these populations included adding information about equity, diversity and inclusion (EDI), breaking down the resource into content that is developmentally staged, and providing parents with knowledge and skills to intervene against suicide. These recommendations are represented in Table 2.

**Table 2:** Recommendations to contextualise the resource for HICs and/or Western countries

Recommendation	Illustrative quote
Developmental staging of guidance	“I don’t know if you can break it down into the different age groups – like when we’re talking about 12-year-olds and 25-year-olds, it’s vast, isn’t it really?” – P20, Ireland
Information about supporting young people with specific mental health needs	“I know [Participant 21] mentioned as well, earlier there, around neurodiversity, and I mentioned as well, mental health problems – is there any particular focus on that, to name it, because if your young person is in that situation, their needs might be slightly different.” – P20, Ireland
Skills to intervene against suicide	“…in terms of extra stuff to add, we have done a lot of work around training, and suicide intervention training, here in Ireland. And empowering our parents by giving them the skill sets around suicide intervention would be a really powerful tool to inform them about – how they can be of practical – and support their young people.” – P20, Ireland
Validating parents’ concerns about social media	“…I’m wondering if initially – like in sort of the introduction, there might be something that says something around, ‘while we are aware that…there can be a negative impact on social media and there’s a lot of concern, we know, from parents…about how it’s being used, yet the fact remains that our kids are using it, and so this guide is basically in response to acknowledging and validating people’s concerns and at the same time, recognising this is where we live, so we have to do something’ – something along those lines.” – P34, USA
Considering equity, diversity and inclusion	“Another element of that is, of course, to make sure that there’s an [EDI] lens and that there are diverse perspectives as well.” – P28, Canada

Edition one of the *#chatsafe Guide for Parents and Carers* was regarded by some participants as being too long and text-heavy, making it feel “*daunting*” as a document (P15, UK). One participant recommended using a reading age analysis tool to simplify the language and “*make it accessible to [all readers]*” (P15, UK). Additionally, many participants supported language translation of the resource to improve accessibility and global relevance, as well as updating emergency services details and support service information for each country.

### A range of marketing and dissemination strategies are needed to reach adults with #chatsafe information

#chatsafe materials and information have traditionally been disseminated as downloadable resources (i.e., standalone, long-form documents) and via social media campaigns, usually targeting young users. However, participants in the present study mostly agreed that this is not the most effective way of reaching parents and carers with #chatsafe information. While some felt that digital channels (e.g., social media, television advertising) would be ideal for sharing suicide prevention information, others felt that in-person settings (e.g., hard-copy resources available in schools) would be more effective.

“*So, here in Brazil, I think that – thinking the ideal situation, I think that the school is very, very important too for that. But we don’t have here adults [there], but we have our adults inside the health system. So I thought that here in Brazil, it’d be more productive to think that, and to think on television and on social media. Because we have a lot of adults using social media here*.” – P36, Brazil.

“*I don’t think social media would be the good way for parents, necessarily. I think through the schools might be a good way because they read stuff from the schools, usually. I’m not saying don’t do social media, but I wouldn’t rely exclusively on that, so you probably want to have multiple avenues for marketing – so that would be one.”* – P34, USA.

In terms of format, many suggestions were put forward to produce short-form and interactive content that may be more effective at engaging large audiences and developing wider awareness and interest in #chatsafe resources. Suggestions included one-page fact sheets, short video vignettes demonstrating concepts from the resource, social media campaigns for adults, and cartoons, with a focus on materials that can be “*shareable in an easy way*” (P4, Brazil).

Participants from mental health organisations expressed a strong desire to market and disseminate the full resource widely using a range of digital and physical avenues (e.g., social media campaigns, television and radio advertisement, flyers at schools and medical centres), as well as an appetite for conducting activities such as webinars and conferences to reach industry professionals. Further to this, participants from Nigeria described how the resource could be “*very useful to us*” (P33, Nigeria) in outreach projects aiming to improve mental health literacy in schools and communities.

## Discussion

The objective of this study was to update and contextualise the #*chatsafe Guide for Parents and Carers* for audiences in 15 countries worldwide. The original resource was developed in Australia to support parents and carers to feel confident talking to their young person about self-harm, suicide and social media [39]. To improve the relevance of this resource for parents and carers in broader global contexts, the present study sought to understand the concerns held by parents, carers and suicide prevention professionals around the world about these topics and to explore the extent to which a resource such as #chatsafe would be helpful within their communities.

Findings from this study highlight the universal nature of concern and complexity of self-harm, suicide, and social media, particularly when it comes to young people. Despite spanning 15 different locations across higher and lower economic regions, all participants in this study acknowledged challenges and varying degrees of (dis)comfort with communication about these topics. Participants raised commonly heard concerns related to talking about self-harm and suicide, such as adults fearing that they will put ideas in a young person’s mind [40], a sense that these topics were ‘too big’ for themselves, let alone young people, and that sometimes these topics were better left unspoken [41]. These beliefs were underscored by country-related factors and cultural norms that shaped participants’ views.

Given the high degree of stigma towards mental health and suicide in many of the countries included in this study, it is not surprising that self-harm and suicide were not widely discussed topics between adults and young people. Participants seemed aware that young people were more likely to have these conversations with other young people, and that it was adults that were less likely to communicate openly about suicide, which echoes generational differences in mental health awareness and openness about these topics [42]. It might be expected that higher avoidance and stigma towards these topics is present in countries where suicide is still classified as a criminal act [43], which is consistent with the perspectives of some participants in this study. However, even in countries that regard themselves as more progressive in their mental health literacy (e.g., Canada, Ireland, UK, USA), the pervasive myth among adults that it is unsafe to talk about suicide prevailed [44]. These beliefs impacted the views participants held about the safety of self-harm- and suicide-related communication online.

Participants in this study reported that concerns held about self-harm or suicide by themselves or other adults in their country were amplified when considering social media in two ways. First, some participants raised concerns about the time young people spend online and the impact social media has on mental health more generally. This reflects a wealth of correlational research exploring the rise in suicidal behaviour in conjunction with increased digital media use by young people [9, 10, 12]. Second, almost all participants described concerns that young people’s exposure to sensitive content online may normalise or promote self-harm or suicide. Exposure to self-harm- or suicide-related content has, in some cases, been shown to be associated with increased suicide ideation or urges to engage in self-harm in young people [45, 46], so the worry related to the impact of graphic, unsafe, or distressing content is warranted. However, rather than this concern spanning all suicide-related communication, it is the quality and quantity of media content that needs to be considered [42]. For instance, helpful suicide prevention messaging on social media has been shown to increase young people’s ability to communicate safely about suicide and provide support to peers [29, 30], highlighting the possibility of using social media to provide support to young people at risk of suicide.

A core concern for participants in this study related to suicide-related ‘trends’ or social media challenges, and the role of sensationalist media reporting (e.g., [47]) in exacerbating panic and unwanted attention towards these events. Despite mixed evidence supporting the true existence of suicide games or social media challenges [48], adults remain fearful of their potential for harm. In the present study, these concerns were situated within broader worries regarding the vast and unregulated nature of social media content, and that young people were more likely to communicate online about sensitive topics like suicide late at night, in closed groups, and in ways that are largely invisible. The strength and presence of these concerns across participants from all countries included in the present study underscore the need for promoting digital literacy and suicide prevention psychoeducation for young people and adults around the world.

### Implications

This was the first study to explore the specific concerns held by parents, carers and suicide prevention professionals about social media and suicide-related communication, with a view to providing relevant and reliable information to support them to safely discuss these topics with young people. Despite the different contexts through which concerns about these topics manifested, participants’ diverse perspectives and experiences represented a set of unified ideas; (1) efforts to support the safety of young people (both online and offline) are important and worthwhile, (2) systems and stakeholders (e.g., families, schools, social media companies, governments) can and should be doing more to protect young people from online harms, and (3) parents and carers play a critical role in this effort, but cannot do it alone.

Findings from this study support literature highlighting that conversations about self-harm and suicide with young people are challenging and often uncomfortable [49, 50]. While it is reasonable to expect that these conversations will never feel easy or comfortable for most people, initiatives such as #chatsafe seek instead to ensure that members of the community feel empowered and able to talk about self-harm and suicide in a way that feels safe and protective. Participants in the present study reported finding resources such as #chatsafe useful in the sense that they are a safe entry point for general mental health literacy and can provide suicide prevention psychoeducation in an accessible format. Indeed, there is little guidance for parents and carers on how to talk to their young person about self-harm and suicide. These findings suggest that embedding sensitive topics like self-harm or suicide into conversations about online safety or social media seemed to make them more approachable. Encouraging open communication between parents and their young person about social media and mental health more generally also supports findings from previous studies that attribute positive health behaviour and meaningful conversations with online parent-child interactions [51, 52]. Such findings highlight the potential for digital literacy programs to make conversations about self-harm and suicide less abstract and more relevant to anyone who spends time online. It also supports the global and cross-disciplinary efforts required to increase online safety with a greater understanding of its potential to decrease suicide risk among young people [53].

There was a general sense that countries with higher rates of mental health literacy and lower stigma (often High-Income, Western countries) would benefit from more specific information about the needs and risks for young people in specific age groups or with particular mental health considerations, and training to intervene against suicide. This assumes a pre-existing level of open conversation about mental health topics or knowledge in these communities. Conversely, participants from countries that described lower mental health literacy, higher mental health stigma, and a greater degree of hierarchy within family structures (often Low- and Middle-Income and/or non-Western countries) indicated a need for more foundational information to improve open communication among families and reduce societal mental health stigma as pre-requisite information before safe online communication about these topics can be seen as an achievable and relevant objective. Not only are young people from LMICs overrepresented in global suicide statistics, but these countries are underrepresented in literature on the development, implementation and/or evaluation of suicide prevention initiatives [54], and it is often assumed that suicide prevention efforts from other parts of the world can simply be applied to these regions. In developing or adapting suicide prevention resources, sociocultural factors that may impact relevance of the final output to various population groups should be considered and communicated transparently to maximise the likelihood of making a meaningful impact [55].

Despite being initially developed for young Australian audiences, #chatsafe resources were perceived by participants in the present study as a valuable tool to improve suicide literacy and promote help-seeking across different generations and social contexts, bringing the beliefs of adults and older generations in line with the support needs of young people. The present study builds upon the existing #chatsafe evidence base by strengthening our understanding of how topics like self-harm, suicide, and social media are/are not discussed among families in various countries around the world. Furthermore, this has been the first study to determine the relevance, usefulness and limitations of the *#chastafe Guide for Parents and Carers* according to its end users, in a wide range of cultural and social contexts. As this is the most highly accessed #chatsafe resource, the present study represents an invaluable and translational activity that is expected to increase uptake of the #chatsafe intervention worldwide, improving safe communication about self-harm and suicide both in-person and online.

### Limitations

While this study provides valuable insights into the concerns held by parents, carers and suicide prevention professionals about social media and suicide, there are some limitations that may impact the interpretation of findings. First, this study utilised a purposive and snowballing recruitment strategy to ensure that relevant organisations and individuals were able to speak to these issues and to the use of #chatsafe more broadly. While this approach elicited relevant perspectives, particularly in relation to the second research question, it meant that many participants were known to the research team, which may have led them to be less critical of the resource than they may otherwise have been. It also meant that only the perspectives of individuals who felt equipped and comfortable to discuss these topics, and able to articulate their concerns, were included. As such, adults who hold a strong belief that suicide should not be discussed with young people are not represented in this study. Second, the #chatsafe resources developed as an outcome of this study were based on a set of evidence-informed guidelines that were developed via a robust Delphi process [35]. Prioritising fidelity to the original evidence base limited scope to fully adapt the guide for parents and carers for each country, even if participant feedback contradicted the original evidence. This was also the case in previous #chatsafe globalisation activities [34]. To partly address this limitation in the current study, the 15 contextualised versions of this resource included country-specific information that spoke to the issues raised by participants from the relevant country (see: [38]). To truly reflect the specific culture and needs of local communities around the world, a next step would be a rigorous adaptation process that repeats the Delphi methodology in each country or region. Such a study is currently underway in New Zealand, which will convene local expert panels (one of young people; one of other stakeholders; 50% Māori on both) to adapt #chatsafe for indigenous rangatahi and young people in New Zealand [56]. Similar activities could be conducted elsewhere in partnership with local communities and will result in guidance that best reflects the online safety and help-seeking landscape in culturally sensitive ways that are most meaningful for individuals in those locations.

## Conclusion

Acknowledging the reality of an increasingly digital world, the quality of online communication about self-harm and suicide must be considered as an important component of youth suicide prevention. Efforts that lead to safer online communities and communication about these topics are needed, and the role of parents and carers in supporting these outcomes cannot be overlooked. The present study has found that parents, carers and suicide prevention professionals around the world share similar concerns about unsafe online trends and young people’s ability to safely navigate online spaces, while also feeling that adults are ill-equipped to communicate about self-harm, suicide or social media with young people. The *#chatsafe Guide for Parents and Carers* presents a valuable opportunity to empower parents and carers with the tools to better support young people on topics of self-harm, suicide and social media, and may be useful in addressing prominent concerns, stigma and misinformation among a range of populations. While there is a clear and present need for country- and culture-specific suicide prevention resources, participants in the present study emphasised the necessity and timeliness of resources like #chatsafe in improving young people’s online safety and fostering more open and effective communication about self-harm and suicide among families around the world.

## Data Availability

The datasets generated and/or analysed during the current study are not publicly available due to inability to sufficiently anonymise transcripts, but are available from the corresponding author on reasonable request.

## Abbreviations

EDI: Equity, Diversity and Inclusion
HIC/s: High-Income Country/Countries
LMIC/s: Low- and Middle-Income Country/Countries
NGO: Non-Government Organisation/s
USA: United States of America
UK: United Kingdom
